# Clinical Observation: Congenital Absence of the Left Portal Vein in a Patient Undergoing Hepatic Resection

**DOI:** 10.1155/1997/18965

**Published:** 1997

**Authors:** C. K. Charny, P. Ling, J. Botet, L. H. Blumgart

**Affiliations:** 1 Department of Surgery The New York Hospital New York N.Y. 10021 USA; 2 Department of Surgery Memorial Sloan-Kettering Cancer Center New York N.Y. 10021 USA; 3 Department of Radiology Memorial Sloan-Kettering Cancer Center New York N.Y. 10021 USA

## Abstract

Congenital absence of the left portal vein is a rare vascular anomaly with a reported prevalence varying from one in 62 to one in 507 cases. A patient admitted for recurrent cholangitis secondary to extensive dilation of the left biliary ductal system associated with Caroli's Disease was determined by preoperative dynamic CT to have an excessively large right portal vein and no left portal vein. The surgeon must be aware of any variations in portal vascular anatomy in patients undergoing hepatic resection in order to prevent potentially fatal postoperative complications.


*HPB Surgery*, 1997, Vol. 10, pp. 323–327
Reprints available directly from the publisher
Photocopying permitted by license only

© 1997 OPA (Overseas Publishers Association)
Amsterdam B.V. Published in The Netherlands
by Harwood Academic Publishers
Printed in India

### *Case Report*

### Clinical Observation: Congenital Absence of the Left Portal Vein in a Patient Undergoing Hepatic Resection

C. K. CHARNY[a], P. LING[b], J. BOTET[c] and L. H. BLUMGART[b]

[a]*Department of Surgery, The New York Hospital, New York, N.Y. 10021;* [b]*Department of Surgery, Memorial Sloan-Kettering Cancer Center, New York, N.Y. 10021;* [c]*Department of Radiology, Memorial Sloan-Kettering Cancer Center, New York, N.Y. 10021*

*(Received 10 August 1996)*

**Congenital absence of the left portal vein is a rare vascular anomaly with a reported prevalence varying from one in 62 to one in 507 cases. A patient admitted for recurrent cholangitis secondary to extensive dilation of the left biliary ductal system associated with Caroli's Disease was determined by preoperative dynamic CT to have an excessively large right portal vein and no left portal vein. The surgeon must be aware of any variations in portal vascular anatomy in patients undergoing hepatic resection in order to prevent potentially fatal postoperative complications.**

*Keywords:* Liver resection, importance of portal vein anatomy

### REPORT OF A CASE

A 33-year-old woman was referred to Memorial Sloan-Kettering Cancer Center for evaluation and treatment of recurrent cholangitis following multiple operations carried out in an attempt to remove a choledochus cyst. At age 8, the patient underwent bypass drainage of a choledochus cyst by Roux-en-Y choledochojejunostomy. Twenty-two years later, the choledochus cyst was partially resected with subsequent Roux-en-Y hepaticodochojejunostomy. Subsequent to hospitalization for bacteremia, operative dilation of both the right and left hepatic ducts was performed. She continued to have attacks of recurrent cholangitis and bacteremia and further operation was considered necessary.

On admission, review of a CT scan revealed extensive cystic dilation of the left intrahepatic ductal system consistent with Caroli's Disease (Fig. 1). The preoperative dynamic CT scan also revealed an excessively large right branch of the portal vein and complete absence of the left branch of the portal vein (Fig. 2). A HIDA scan showed no evidence of biliary obstruction. Formal left hepatectomy with biliary-enteric anastomosis was planned.

At operation, the left liver was atrophic and contained multiple cysts. The left intrahepatic ductal system was dilated in a cystic manner down to an anastomosis to a Roux-en-Y loop of

Corresponding author: Leslie H. Blumgart, M.D., Chief, Hepatobiliary Service, Department of Surgery, Memorial Sloan-Kettering Cancer Center, 1275 York Avenue, New York, N.Y. 10021, Telephone: 212-639-5526 or Fax: 212-794-5852.

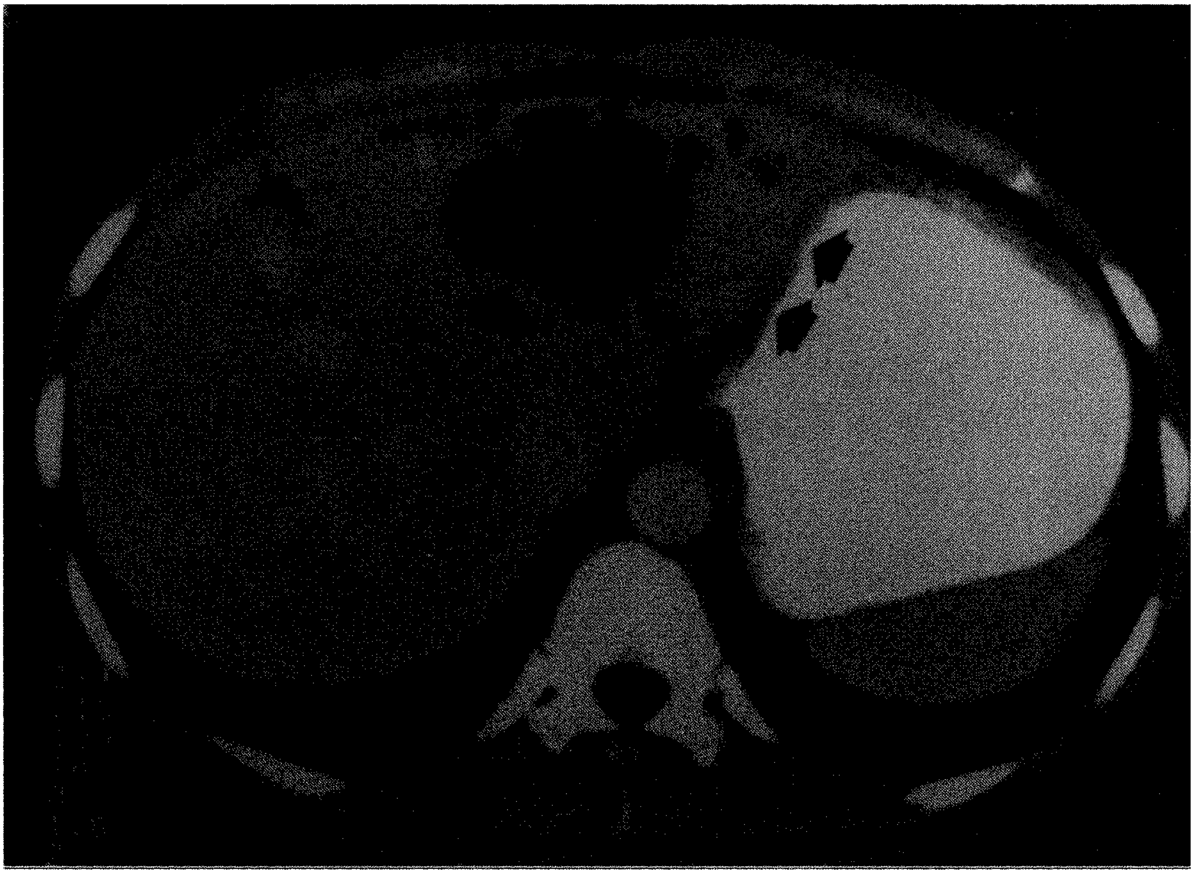

FIGURE 1 Caroli's Disease as demonstrated by the large intrahepatic cyst-like dilatation of the left ducts. Note the small left hepatic lobe (double arrowheads) which is also seen in Figure 2.

jejunum, just proximal to which there was a remnant of a choledochus cyst into which the right hepatic duct entered. The caudate lobe was normal in size and was supplied with portal blood via caudate branches arising from the portal vein. The right branch of the portal vein was notably enlarged, to supply portal blood to both the right and left hepatic lobes in the absence of the left branch of the portal vein.

Intraoperative ultrasound revealed no cystic dilations within the right hepatic lobe. The cystic remnant at the right hepatic orifice was excised. Total left hepatectomy was performed and absence of the left portal vein was confirmed. The right hepatic ducts were anastomosed to a Roux-en-Y loop of jejunum.

Postoperatively, the patient had low grade fevers. A CT scan revealed fluid collections adjacent to the right hepatic Remnant. CT guided percutaneous drainage yielded 200 cc of bilestained pus. The patient was started on a course of intravenous antibiotics and was discharged from the hospital ten days later. She remains symptom free and a HIDA scan perfomed at nine months followup was normal.

### COMMENT

Congenital absence of the main left branch of the portal vein has been documented in several reports [1–6]. Anatomical studies by Couinaud

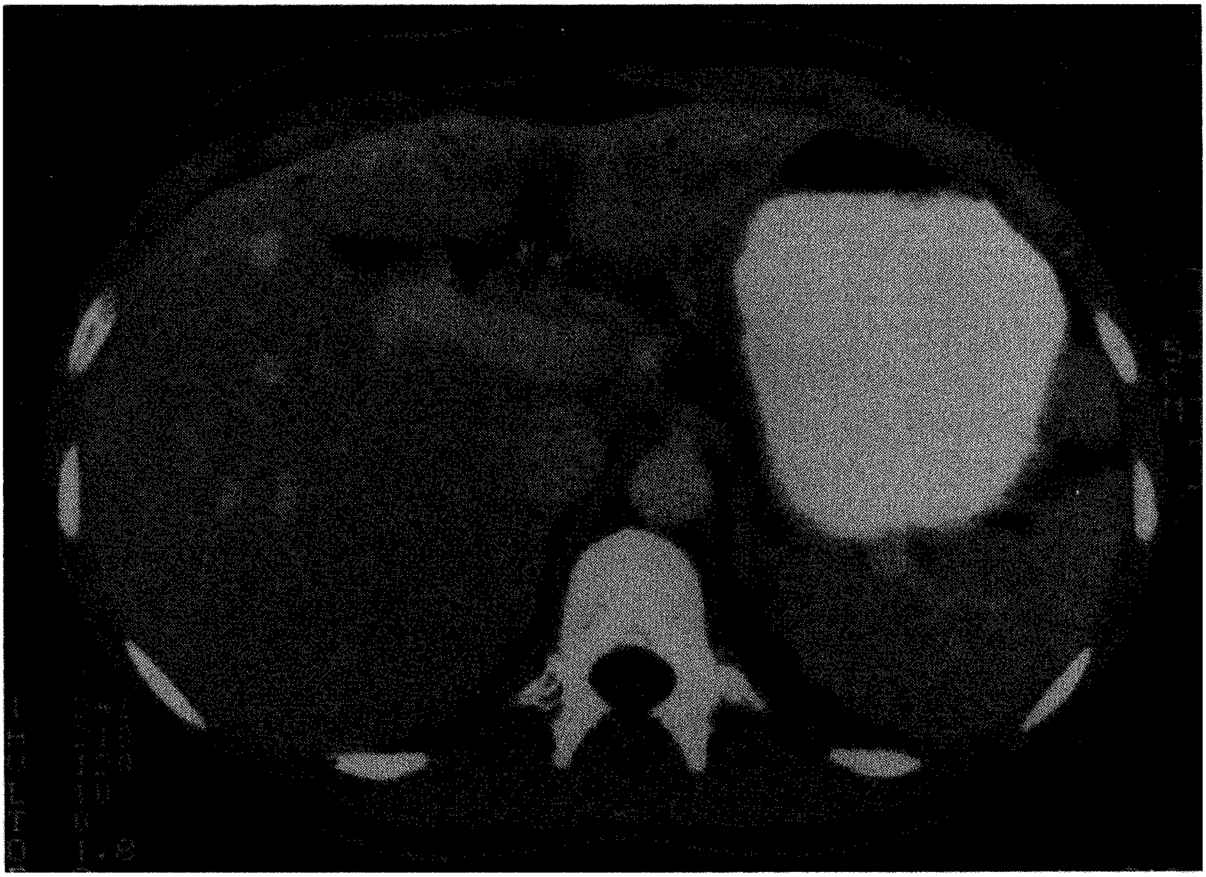

FIGURE 2 Dynamic CT of liver demonstrating the dominanat right portal vein (large arrowhead). No left portal vein is demonstrated. The hepatic arteries are well visualized.

and Abosson-Voyeme reported this vascular anomaly in one in 103 cases and one in 62 cases, respectively [1, 3]. A prospective ultrasound study by Atri *et al.* Reported a prevalence of one in 507 cases [5]. It is interesting to note that in Couinaud's study, the hepatic artery and biliary duct distribution was always normal.

The surgical implications of absence of the left portal vein are significant. In the case described by Kohn *et al.*, ligation of the right branch of the portal vein during right hepatectomy in a patient without a left branch of the portal vein resulted in complete portal vein thrombosis with subsequent postoperative death due to multiple organ failure and sepsis. [2]. Thus, the surgeon must be aware of the existence of any variations in portal vein anatomy either preoperatively or intraoperativey. This concept was discussed by Couinaud in a recent monograph. [1]. CT portography and ultrasonography are both suitable techniques by which to make this determination. Variations include main portal vein trifurcation, a right posterior sectoral branch originating from either the main or left portal vein, and absence of the main right or main left branch of the portal vein. [5].

### *References*

[1] Couinaud, C. (1989). Surgical anatomy of the liver revisited. Paris pers. ed.

[2] Kohn, M. K., Ahmad, H., Watanapa, P., Jalleh, R. P. and Habib, H. A. (1994). Case report: Beware the anomalous portal vein, *HPB Surgery*, **7**, 237–40.
[3] Abosson-Voyeme, A. K. (1982). La segmentation hepatique en romedenistometrie, Paris These 3e cycle.
[4] Fraser-Hill, M. A., Atri, M., Bret, P. M., Aldis, A. E., Illescas, F. F. and Herschorn, S. D. (1990). Intrahepatic portal venous system, Variations demonstrated with duplex and color doppler US, *Radiology*, **177**, 523–6.
[5] Atri, M., Bret, P. M. and Fraser-Hill, M. A. (1992). Intrahepatic portal venous variations, Prevalence with US, *Radiology*, **184**, 157–8.
[6] Takashi, K., Kazuki, I. and Seiji, K. (1987). CT demonstration of bizarre intrahepatic portal branch, *J. Comput. Tomogr.*, **11**, 365–7.

**Invited commentary** to: "Congential absence of the left portal vein in a patient undergoing hepatic resection".

### INVITED COMMENTARY

The message of this paper is important: be aware of possible anatomic variants when performing resection of the liver. Variations in portal venous or biliary anatomy are infrequent but may lead to disastrous consequences if unrecognized at the time of surgery. Thus, as discussed in this paper, ligation of the right branch of the portal vein, in the absence of a left branch, may lead to postoperative death [1]. Likewise, ligation of the left portal branch during left hepatectomy would lead to problems in a patient with anomalous take-off of the right anterior branch from the left portal vein [2, 3]. Right hepatectomy in the absence of a left hepatic duct has led to hepatic transplantation [4]. Following left hepatectomy, bile drainage may be jeopardized if there is anomalous drainage of the right duct into a dominant left duct [5]. It should be emphasized that a portal venous or biliary anomaly may be present without anomalies in the other system.

The way to avoid problems due to congenital abnormalities is, of course, to diagnose variants before hepatic transection. With respect to the portal venous anatomy, variations can be demonstrated on ultrasound combined with colour duplex ultrasonography [2]. In the present paper, the congenital absence of the left portal vein was observed on dynamic CT. However, our experience is that CT is not a reliable method for delineating portal venous anomalies, and this is true also for computed tomographic arterial portography (CTA). A reliable demonstration of the intrahepatic portal venous system could be made with standard angiography. Such a procedure is not part of the routine work-up before liver resection and is not warranted solely for demonstrating the vascular anatomy.

With respect to intrahepatic biliary anomalies, ultrasound, CT and magnetic resonance imaging (MRI) are unable to give reliable findings. The situation is going to be improved quite rapidly, however, because three-dimensional imaging with CT or MRI is expected to give good intrahepatic pictures of both the portal and biliary trees. Endoscopic retrograde cholangiopancreatography should not be used preoperatively solely for delineating the biliary anatomy.

Awaiting reliable and readily available imaging tools, I would suggest the following routine. Preoperative and/or intraoperative ultrasound should be performed in order to disclose portal venous anomalies. Major liver resection should be preceded by dissection and definition of the vascular and biliary structures in the liver hilum, which may be wise even if ultrasonography indicates normal anatomy. If an anomaly of the biliary tree is suspected or cannot be ruled out, intraoperative cholangiography should be performed. Hepatic resection with the Couineaud technique [6, 7] should not be performed unless it has been demonstrated that the anatomy is suitable for this procedure.

### *References*

[1] Kohn, M. K., Ahmad, H., Watanapa, P., Jalleh, R. P. and Habib, H. A. (1994). Case report: Beware the anomolous portal vein, *HPB Surgery*, **7**, 237–240.
[2] Atri, M., Bret, P. M. and Fraser-Hill, M. A. (1992). Intrahepatic portal venous variations: prevalence with US, *Radiology*, **184**, 157–158.

[3] Hiei, K. H., Nimura, Y., Nagino, M., Kamiya, J., Kondo, S., Toyoda, S. and Sibata, Y. (1995). Resection of metastatic liver cancer in a patient with an anomalous intrahepatic portal system: a case report, *Hepato-Gastroenterology*, **42**, 1002–1007.
[4] Thompson, E. C., Grier, J. F., Gholson, C. F., and McDonald, J. C. (1995). A critical review of the Couinaud technique of hepatic resection, *Archives of Surgery*, **130**, 553–556.
[5] Healey, J. E. and Schroy, P. C. (1953). Anatomy of the biliary ducts within the human liver: analysis of the prevailing pattern of branching and the major variations of the biliary ducts, *Archives of Surgery*, **66**, 599–616.
[6] Couinaud, C. M. (1985). A simplified method for controlled left hepatectomy, *Surgery*, **97**, 358–361.
[7] Lazorthes, F., Chiotasso, P., Chevreau, P., Materre, J.-P. and Roques, J. (1993). Hepatectomy with initial suprahilar control of intrahepatic portal pedicles, *Surgery*, **113**, 103–108.

Karl-G Tranberg Department of Surgery
Lund University
Lund
Sweden

